# Insight into molecular profile changes after skeletal muscle contusion using microarray and bioinformatics analyses

**DOI:** 10.1042/BSR20203699

**Published:** 2021-01-19

**Authors:** Na Li, Ru-feng Bai, Chun Li, Li-hong Dang, Qiu-xiang Du, Qian-qian Jin, Jie Cao, Ying-yuan Wang, Jun-hong Sun

**Affiliations:** 1 School of Forensic Medicine, Shanxi Medical University, Jinzhong 030604, Shanxi, China; 2 Key Laboratory of Evidence Science, China University of Political Science and law, Beijing, China; 3 Collaborative Innovation Center of Judicial Civilization, Beijing, China

**Keywords:** bioinformatics analysis, gene expression profile, microarray, Skeletal muscle healing, wound age

## Abstract

Muscle trauma frequently occurs in daily life. However, the molecular mechanisms of muscle healing, which partly depend on the extent of the damage, are not well understood. The present study aimed to investigate gene expression profiles following mild and severe muscle contusion, and to provide more information about the molecular mechanisms underlying the repair process. A total of 33 rats were divided randomly into control (*n*=3), mild contusion (*n*=15), and severe contusion (*n*=15) groups; the contusion groups were further divided into five subgroups (1, 3, 24, 48, and 168 h post-injury; *n*=3 per subgroup). A total of 2844 and 2298 differentially expressed genes (DEGs) were identified using microarray analyses in the mild and severe contusions, respectively. From the analysis of the 1620 coexpressed genes in mildly and severely contused muscle, we discovered that the gene profiles in functional modules and temporal clusters were similar between the mild and severe contusion groups; moreover, the genes showed time-dependent patterns of expression, which allowed us to identify useful markers of wound age. The functional analyses of genes in the functional modules and temporal clusters were performed, and the hub genes in each module–cluster pair were identified. Interestingly, we found that genes down-regulated at 24-48 h were largely associated with metabolic processes, especially of the oxidative phosphorylation (OXPHOS), which has been rarely reported. These results improve our understanding of the molecular mechanisms underlying muscle repair, and provide a basis for further studies of wound age estimation.

## Introduction

Skeletal muscle tissues, which are widely distributed throughout the body, account for 30–50% of total body mass [1,2]. These tissues have a relatively superficial location and are frequently damaged in daily life. Despite skeletal muscle having a unique ability for rapid and extensive repair in response to damage, major injuries result in dysfunctional and incomplete functional repair, where the injured muscle may be replaced by connective tissue, thus impairing muscle function [3–5]. Studies from numerous groups have demonstrated that muscle tissue repair is a complex phenomenon involving degeneration, inflammation, regeneration, and fibrosis, which are continuous processes with significant overlap [6,7]. Muscle injury models are being developed to improve understanding of how tissues respond to injuries caused by mechanical stress [8].

Although many basic characteristics of wound healing after injury have been described and explained, the complex interactions and interrelationships of multiple genes and regulatory pathways during the process are still poorly understood. Moreover, outcomes differ by injury severity: successful repair or inadequate regeneration (fatty degeneration and formation of fibrotic scar tissue) may be observed [9]. However, relatively less research aims to explore the cellular responses to muscle injury of different severities, knowing less about shared and divergent molecular mechanisms among damage degrees during muscle repair process. These issues emphasize the importance of investigating the wound-healing response to mechanical muscle damage at different levels, to improve treatment of major injuries.

Muscle contusion is another highly frequent event in violent cases, and forensic examiners are often required to detect skeletal muscle injury and determine wound age (time between the infliction of a wound and time of death). The difficulty of estimating injury occurrence time largely depends on the vital reaction after injury [10,11]. The vital reaction and wound age are among the most important and well-studied areas of forensic research, and many studies are being conducted with the aim of identifying markers of wound healing. Barington et al. and Yu et al. showed that the infiltration of inflammatory cells [12–14] and expression levels of regulatory mediators (cytokines, growth factors etc.) [15,16] show a time-dependent response after injury, and could be useful for wound age estimation.

Precise wound age estimation remains difficult for forensic pathologists worldwide, due to limited understanding of the skeletal muscle repair process, particularly at the molecular level. Following muscle damage, a complex process occurs involving various cellular and molecular responses [17], and coordination between inflammation and regeneration. Injured muscles evaluated in forensic practice often differ in the degree of damage; differences in outcomes raise the question of whether a similar damage repair process is involved in injuries varying in severity. Thus, elucidating the gene expression profiles associated with the repair process after skeletal muscle injury is important, with consideration of the degree of contusion, as well as the functions and interactions of differentially expressed genes (DEGs).

In the present study, because the basic physiological response is similar between humans and animals, animal models of muscle contusion (mild and severe) were developed to investigate the muscle repair process in a controlled and reproducible manner. Additionally, transcriptome-based microarray technology, a high-throughput method, was used as an efficient way to identify DEGs involved in various biological processes [18]. The goal was to lay a foundation for elucidating the molecular mechanisms underlying muscle repair of different damage degrees.

## Methods

### Animal model of skeletal muscle contusion

All procedures were performed according to the ‘Guiding Principles in the Use and Care of Animals’ (NIH publication no. 85-23, revised 1996) and were approved by the Institutional Animal Care and Use Committee of Shanxi Medical University, China (rat batch number: SCXK [Jin] [2009-0001]). Animals received humane care conforming to the principles of the ‘Guide for the Care and Use of Laboratory Animals’ protocol, published by the Ministry of the People’s Republic of China (issued 4 June 2004). The laboratory personnel were permitted to conduct the present study by the above committee after attending and completing training on how to use experimental animals ethically.

In total, 33 Sprague–Dawley male rats, aged 10–12 weeks and weighing 250–300 g, were placed in a cage with rat chow and water, under a 12-h light–dark cycle in a temperature-controlled room (22–24°C) with a relatively humidity of 40–60%. All rats were randomly divided into three groups and treated according to skeletal muscle contusion rat models that have been described previously [19]:

1. Control group (*n*=3): no treatment before sampling;
2. Mild contusion group (*n*=15): a 500-g counterpoise weight (diameter, 0.8 cm) fell from a height of 15 cm through a clear Lucite tube on to the thigh muscles of the right posterior limb, with an energy of ∼0.74 J (calculated by E = mgh) transferred to the limb;
3. Severe contusion group (*n*=15): a 500-g counterpoise weight (diameter, 1.1 cm) fell from a height of 50 cm through a clear Lucite tube on to the thigh muscles of the right posterior limb, with an energy of ∼2.45 J transferred to the limb (*n*=15).

The animals of the mildly and severely contused groups were further divided into five subgroups (1, 3, 24, 48, and 168 h post-injury; *n*=3 per subgroup). Before contusion, the 30 treated rats were anesthetized with pentobarbital; their right posterior limbs were depilated with an agent (Nair; Carter Wallace, New York, NY, U.S.A.), and they were placed in a supine position on an experimental table. After injury, the rats were transferred into clean cages with food and water.

### Tissue preparation

The rats in the mild and severe contusion groups were killed at 1, 3, 24, 48, 168 h post-injury with a lethal dose of pentobarbital (350 mg/kg body weight, intraperitoneal injection). For each rat, approximately 100 mg of muscle was sampled from the wound site and divided into two equal parts. For the control rats, specimens were harvested from the same site after anesthetization with an overdose of pentobarbital. All muscle samples for microarray analysis were frozen immediately with liquid nitrogen.

### Microarray

Total RNA was extracted from the skeletal muscle specimens (approximately 50 mg each) using a reagent (RNAiso Plus 9108; Takara Bio, Shiga, Japan), following the manufacturer’s instructions. The concentration (ng/ml) and purity of the freshly extracted total RNA were measured using a microplate reader (Infinite M200 Pro; TECAN, Zurich, Switzerland), and the integrity of total RNA was determined with the 2100 Bioanalyzer and RNA 6000 Nano assay kit (Agilent Technologies, Palo Alto, CA, U.S.A.). Only RNA with an OD260:OD280 ratio in the range 1.8–2.2 and RNA integrity > 7.0 were used in the microarray analysis. Total RNA was used to synthesize sense-strand cDNA (sscDNA) using the WT Expression Kit (Ambion, Austin, TX, U.S.A.). Then, the sscDNA samples were fragmentated and labeled using another kit (GeneChip® WT Terminal Labeling Kit; Affymetrix®, Santa Clara, CA, U.S.A.) and hybridized to array plates (Gene 2.0 ST Arrays; Affymetrix®). Finally, the arrays were scanned using the GeneChip™ Scanner 3000 7G instrument (Thermo Fisher Scientific, Santa Clara, CA, U.S.A.).

### Data analysis

Normalization of data in CEL file format and one-way analysis of variance of the contusion and control groups were performed using Transcriptome Analysis Console software (version 4.0.1; Affymetrix®). mRNAs with a >two-fold change in mean expression compared with the control group (*P*<0.05, false discovery rate [FDR] < 0.05) were considered as DEGs.

The heatmap of overlapping DEGs at all time points was generated using TBtools software (https://github.com/CJ-Chen/TBtools). To evaluate the effect of damage severity and wound age on the expression of genes shared between the mild and severe contusion groups, principal component analysis (PCA) was conducted using SIMCA-P software (version 14.1; Umetrics, Malmö, Sweden). Hierarchical clustering (HCL) was conducted using Morpheus online software (https://software.broadinstitute.org/morpheus).

Weighted gene co-expression network analysis (WGCNA) was conducted using the WGCNA R package. A topological overlap matrix was constructed based on the gene expression by time stage matrix. A module eigengene (ME), the first principal component of the module, was used to represent the expression profile of a given module, and the correlation of each ME with each time point was visualized as a heatmap. We used gene significance (GS) to analyze the correlations between genes and traits, and calculated module membership (MM) to quantify the importance of each gene to its corresponding module. The correlations between genes (in each module) and traits were assessed using GS and MM analyses.

Gene Ontology (GO) annotation and Kyoto Encyclopedia of Genes and Genomes (KEGG) pathway enrichment analysis were performed using David software (version 6.8; https://david.ncifcrf.gov/tools.jsp). Protein–protein interaction (PPI) network was built using the STRING database (version 11.0; https://string-db.org). Cytoscape software was used to visualize the network and screen the hub genes based on their degree of connectivity.

**Figure 1:**
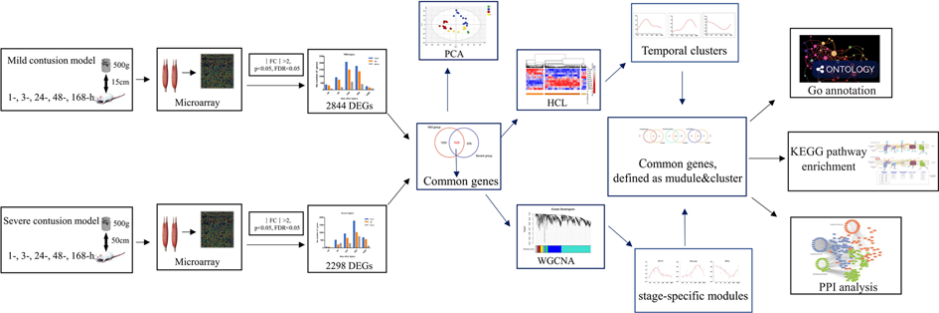
Flow diagram of the experimental and data analysis procedures in the present study

The experimental and data analysis procedures in the present study are summarized in Figure 1.

## Results

### Transcriptional profiles of muscle contusion during the repair process

**Figure 2:**
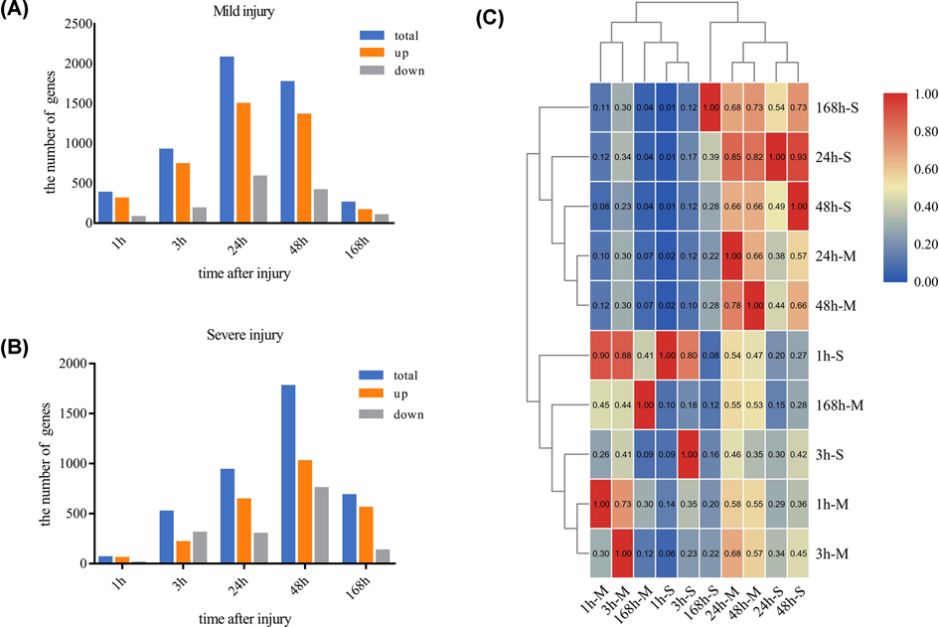
Number of DEGs at all time points during muscle repair; overlapping genes are identified The number of DEGs at each time point for (**A**) mild and (**B**) severe contusion. (**C**) Heatmap of overlapping DEGs in each pairwise comparison. -M, mild; -S, severe.

Comparing with the control group, one-way ANOVA analysis showed that 2844 and 2298 microarray probes (total of 3522 unique probes) exhibited significant changes over the course of healing from mild and severe contusions, respectively. The number of up- and down-regulated DEGs at each time point for the mild and severe contusions were shown in Figure 2A,B. In mild contusion, 2132 probes were up-regulated, 699 probes were down-regulated, and 13 probes changed in different trend overtime; there were 1327 up-regulated probes and 954 down-regulated probes in severe contusion, and 17 differently varied probes. Overall, there was more number of up-regulated than down-regulated DEGs. To investigate the contributions of the extent of damage and wound age to the transcriptome dynamics, pairwise comparisons of DEGS were performed at all time points to identify overlapping DEGs. We produced a heatmap using unsupervised HCL, which showed that adjacent time points with more coexpression genes were clustered together regardless of the degree of damage, indicating commonalities in the mechanisms of transcriptomic repair between mild and severe contusions (Figure 2C).

### Temporal clustering of the genes shared between mildly and severely contused muscle

**Figure 3:**
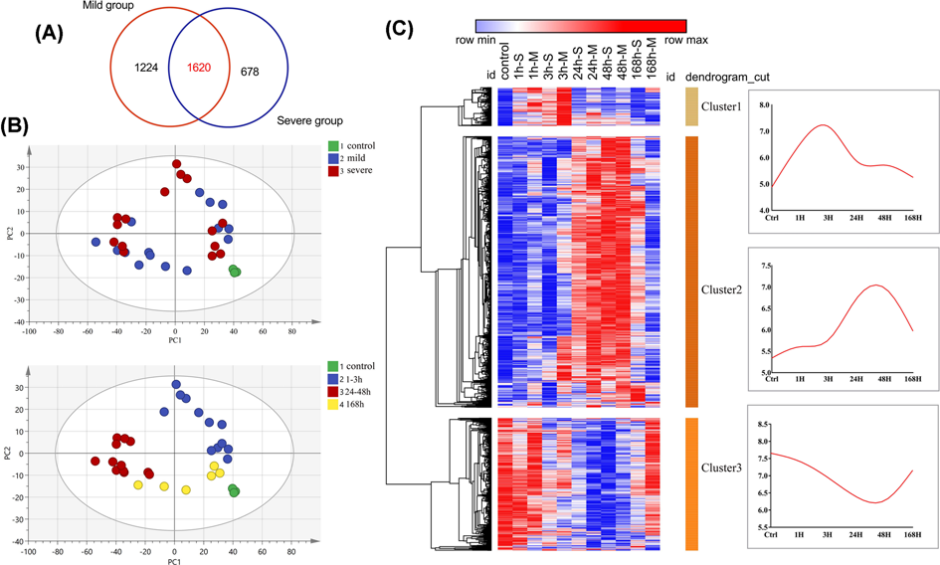
Cluster analysis showing temporal clusters of genes expressed in both mildly and severely contused muscle (**A**) Venn diagram of DEGs between the mild and severe contusion groups. (**B**) PCA results of the 1620 genes shared between the mild and severe contusion groups; samples are colored according to the degree of damage (top) and wound age (bottom). (**C**) Clustered heatmap of all DEGs based on HCL (left: -M and -S indicate mild and severe damage, respectively) and the mean expression of each gene cluster according to wound age (right).

The molecular mechanisms appeared similar and synchronized during the skeletal muscle repair process, and we sought to identify DEGs involved in both mild and severe muscle contusions (Figure 3A). We conducted a PCA of the 1620 shared genes that we identified. In scatter plot of PCA, samples had no distinct rule of damage degrees but displayed a temporal distribution of wound age (Figure 2B). The results revealed that although the transcriptional profiles differed according to the extent of damage, this was not the dominant factor with respect to the changes of coexpressed genes, which were more strongly associated with wound age (Figure 3B).

Having confirmed the existence of similar and synchronized molecular mechanisms, the 1620 genes were divided into three distinct transcriptional clusters using HCL. As shown in the heatmap in Figure 3C, the gene expression patterns of the three clusters diverged over time, and the expression levels of all genes in each cluster exhibited different trends after injury. The expression of genes in clusters 1 and 2 was up-regulated during phases 1–3 h and 24–48 h after injury, respectively, whereas the expression of the genes in cluster 3 was down-regulated after injury, reaching their lowest point at 48 h. These results indicate that the molecular mechanisms underlying cellular functions were more similar during the early (acute) healing phase (within the first 48 h post-contusion), where the different transcriptional profiles after 168 h diverged between the mild and severe contusion groups, with fewer genes exhibiting a significant change in the case of mild contusion.

### Identification of gene coexpression modules associated with wound age based on WGCNA

**Figure 4:**
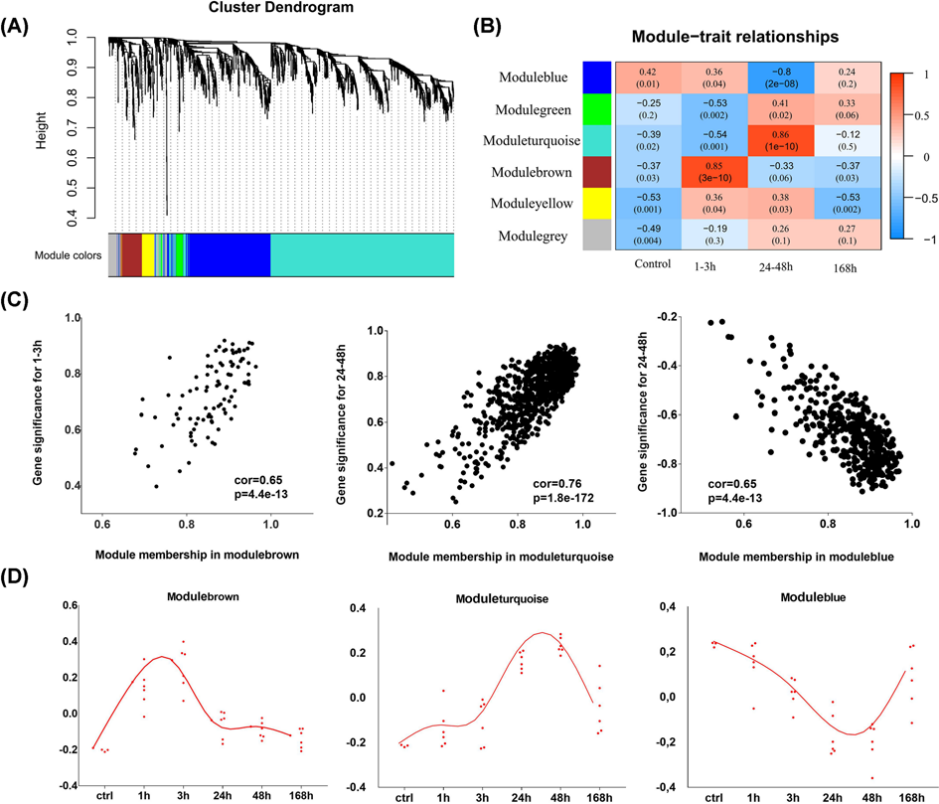
Gene coexpression network analysis for identification of functional modules related to wound age (**A**) Cluster dendrogram of gene modules. (**B**) Correlation analysis of the MEs according to the time since skeletal muscle was contused. (**C**) Correlation between GS and MM for different modules; cor: correlation coefficient. (**D**) Gene expression profiles by wound age for three modules based on their MEs (first principal component of the gene expression analysis); ctrl, control group.

We identified 1620 genes shared between the mild and severe damage groups, which exhibited time-dependent patterns of expression during the (early) acute healing phase (within 48 h post-contusion). We then used WGCNA to explore the coexpression relationships between the DEGs and identify functional modules related to different wound ages. The weighted gene coexpression network was constructed and visualized as an HCL tree with a total of six functional modules, in which genes with similar temporal expression patterns shared certain common biological functions (Figure 4A). The numbers of genes in the modules are listed in Table 1.

**Table 1:** Number of genes in each module

Name of module	Number of genes
Moduleblue	422
Modulebrown	98
Modulegreen	42
Modulegray	87
Moduleturquoise	912
Moduleyellow	59

Then, to identify ‘stage-specific’ modules, the relationships between modules and time stages were calculated. Modulebrown exhibited a significant correlation with the 1–3 h post-contusion time period, while moduleturquoise and moduleblue were strongly related to the 24–48 h post-contusion period (correlation coefficient ≥ 0.8 or ≤ −0.8). The correlation coefficients of the control and 168 h groups were less than 0.6 (Figure 4B). For 168 h, there were a few coexpressed DEGs (only 29 genes) of two damage groups and found no specifically related module. Moreover, the genes involved in these three modules had strong correlations between GS, which is the correlation between a gene and an external trait, and MM, which denotes the importance of a gene to its module; all three modules were closely related to particular time stages (Figure 4C).

**Figure 5:**
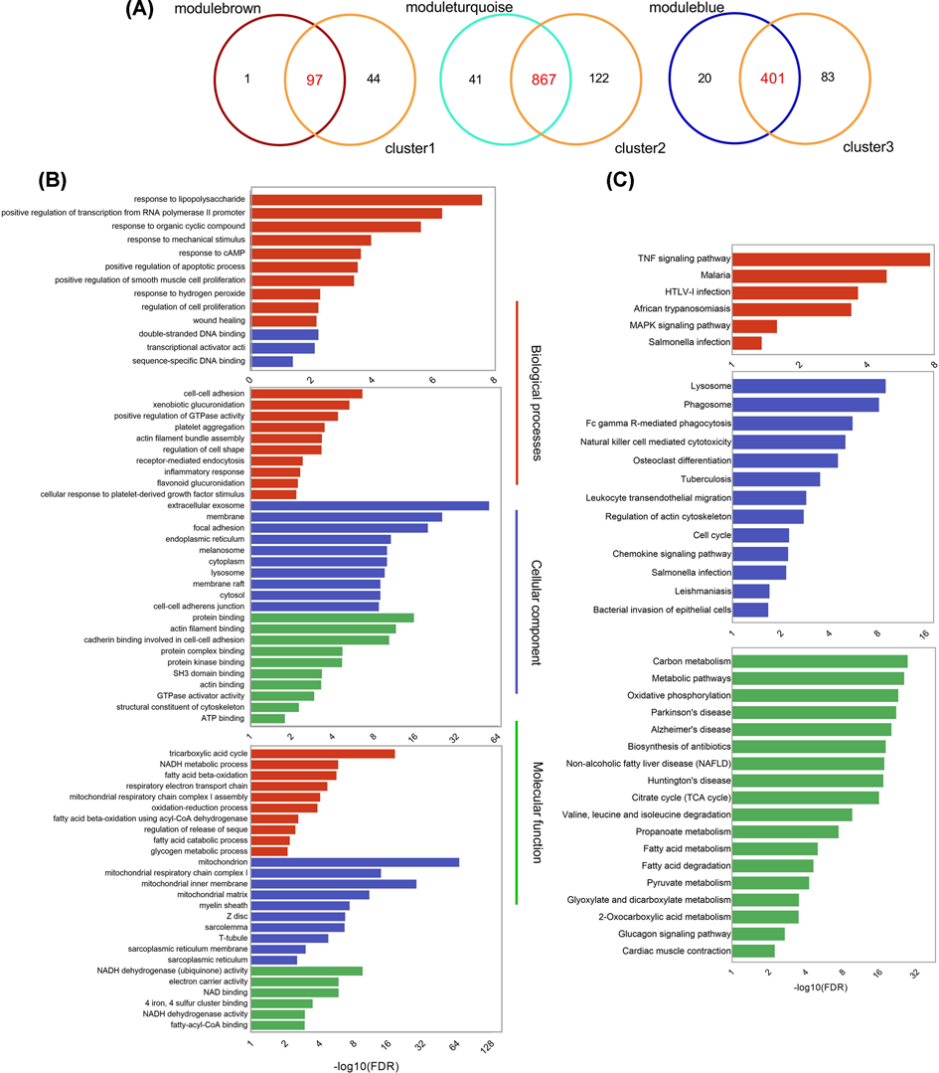
Functional annotations of the shared genes in functional modules and temporal clusters (**A**) Venn diagram of DEGs between modules and clusters. Functional annotation results of genes in each cluster–module pair based on (**B**) GO and (**C**) KEGG pathway enrichment analysis.

We then further analyzed the three modules: stage-specific module expression values (Figure 4D), which were represented by the first principal component of each module (the ME), indicated that modulebrown, moduleturquoise, and moduleblue comprised genes that tended to be overexpressed at 1–3 postinjury, overexpressed at 24–48 h post-injury, and down-regulated at 24-48 h post-injury, respectively. This finding was consistent with those of the temporal clustering using HCL, where the temporal clusters and three corresponding functional modules having largely similar genes. As can be seen from Figure 5A, there were 97 shared genes of modulebrown and cluster1; 867 genes were both in moduleturquoise and cluster2; 401 common genes were between moduleblue and cluster3.

### Functional and pathway enrichment analysis of shared genes between functional modules and temporal clusters

We found that the temporal clusters and three corresponding functional modules having largely shared genes (Figure 5A) showed similar gene expression patterns. We then performed GO classification and pathway enrichment analysis to characterize the distribution of shared genes between clusters and modules according to their biological function. The top ten GO categories and KEGG pathways results (FDR < 0.05) are shown in Figure 5B,C, respectively. Moreover, the enriched KEGG pathways, and the genes involved therein, are listed in Table 2.

**Table 2:** Enriched KEGG pathways of various functional modules and functional module–temporal cluster combinations

Description	Genes	FDR
TNF signaling pathway	*CXCL1, FOS, ICAM1, IL6, PTGS2, SOCS3, JUN, MAP3K8, CXCL2, IL1B, NFKBIA, BIRC3, JUNB*	3.00E-08
Malaria	*HBA-A1, CSF3, ICAM1, SELP, IL6, IL1B, HBB-B1, HBA1, HBB*	1.51E-05
HTLV-I infection	*EGR1, ZFP36, IL1R2, ICAM1, IL6, EGR2, CREM, NFKBIA, FOS, ATF3, JUN, ETS2, MYC, FOSL1*	2.56E-04
African trypanosomiasis	*HBA-A1, ICAM1, IL6, IL1B, HBB-B1, HBA1, HBB*	4.41E-04
MAPK signaling pathway	*DUSP5, MAP3K6, FOS, DUSP2, DUSP1, JUN, GADD45G, MAP3K8, IL1B, MYC, CDC25B*	0.027356
*Salmonella* infection	*CXCL1, FOS, IL6, JUN, CXCL2, IL1B, NOS2*	0.046022
Lysosome	*CLTA, GM2A, LITAF, LGMN, HEXA, HEXB, AP3S1, ACP2, PPT1, CTSA, CLTC, ASAH1, GLB1, SLC11A1, AP1S1, CD68, LAPTM5, GALC, MAN2B1, AP3B1, TCIRG1, CTSS, CD63, M6PR, DNASE2, GNS, CTSL, NPC1, LAMP2, NPC2, CTSD, CTSC, CTSB*	1.58E-09
Phagosome	*RAB7B, MSR1, TUBB2A, RAB5C, TLR2, C1R, ITGB2, TLR4, RT1-DMA, RT1-DMB, ATP6V1B2, TLR6, CALR, CANX, ITGAM, ACTG1, FCGR1A, TUBB5, TUBB6, SCARB1, TUBA1A, TUBA1B, SEC61A1, TCIRG1, ACTB, MRC1, NCF2, NCF4, COLEC12, CTSS, M6PR, ATP6V1A, CYBA, CTSL, CORO1A, LAMP2, SEC61B, FCGR2B, ITGA5, CD14*	9.67E-09
Fc γ R-mediated phagocytosis	*PTPRC, PLD1, VAV3, LYN, PIK3CB, HCK, ASAP1, ARPC4, ARPC5, PRKCD, VAV1, WAS, PRKCB, RAC2, FCGR2B, ARPC3, ARPC2, FCGR1A, CFL1, MAPK3, PIK3R5, INPP5D, SYK*	3.29E-06
Natural killer cell-mediated cytotoxicity	*BID, PTPN6, CD244, VAV3, PIK3CB, ITGB2, VAV1, PRKCB, IFNAR1, HCST, CD48, NRAS, CASP3, RAC2, FYN, MAPK3, FCER1G, SHC1, PIK3R5, IFNGR1, LCP2, TYROBP, SH3BP2, SYK*	1.11E-05
Osteoclast differentiation	*LILRB3A, LILRB3B, SPI1, TGFB1, BTK, LILRB3L, TNFRSF1A, FCGR1A, PIK3R5, IFNGR1, TYROBP, SYK, CSF1R, BLNK, NCF2, PIK3CB, NCF4, SIRPA, IFNAR1, TYK2, CYBA, CYBB, FCGR2B, FYN, MAPK3, TREM2, LCP2*	3.58E-05
Tuberculosis	*BID, RAB5C, IL18, TLR2, ITGB2, TLR4, RT1-DMA, RT1-DMB, TLR6, TGFB1, ITGAM, IRAK4, TNFRSF1A, CASP3, IL10RB, FCGR1A, IL10RA, FCER1G, IFNGR1, SYK, TCIRG1, MRC1, CTSS, LSP1, CORO1A, LAMP2, FCGR2B, MAPK3, CTSD, JAK2, CD14*	3.56E-04
Leukocyte transendothelial migration	*ACTB, VAV3, GNAI3, GNAI2, ACTN4, NCF2, PIK3CB, NCF4, SIPA1, ACTN1, ITGB2, CTNNA1, VAV1, CXCL12, ITGAM, PRKCB, ACTG1, CYBA, EZR, RAC2, PIK3R5, RAP1B, MSN*	0.001492
Regulation of actin cytoskeleton	*FGF7, DIAPH3, ARPC4, ITGB2, ARPC5, IQGAP1, ITGAM, ACTG1, PFN1, EZR, PAK2, RAC2, ARPC3, TIAM1, ARPC2, PIK3R5, MSN, FN1, ACTB, VAV3, ACTN4, PIK3CB, BAIAP2, NCKAP1L, ACTN1, VAV1, WAS, NRAS, ITGA5, CFL1, MAPK3, PDGFRA, PIP4K2A*	0.001869
Cell cycle	*CDK1, YWHAZ, RBL1, YWHAB, TTK, CDK6, CDK4, MCM3, MCM4, TGFB1, MCM5, MCM6, CCNB1, CDKN1A, CCNB2, YWHAH, MAD2L1, HDAC1, PLK1, CDKN2C, PCNA, BUB1, CCNA2*	0.006191
Chemokine signaling pathway	*GNAI3, GNAI2, FGR, PREX1, PF4, CXCL12, CCL7, CCL6, CCL24, RAC2, TIAM1, PIK3R5, SHC1, VAV3, LYN, PIK3CB, HCK, PRKCD, VAV1, WAS, NRAS, GNGT2, CCR5, ARRB2, GNB1, MAPK3, RAP1B, JAK2*	0.006592
Carbon metabolism	*ME3, ECHS1, PGAM2, OGDH, ACSS2, ACAT1, PDHB, HADHA, PGP, ACSS1, TPI1, IDH3G, GOT1, MCEE, PDHA1, SUCLA2, GPT2, FH, ALDH6A1, ACADM, ACO2, ACADS, SUCLG1, CS, IDH3B, DLAT, PFKM, FBP2, IDH3A, SDHA, SDHB, SDHC, SDHD, GPT, PCCB, MDH2, PCCA, MDH1*	3.48E-27
Metabolic pathways	*PGAM2, OGDH, ACSS2, COX5B, HIBADH, PDHB, CMBL, ACSS1, PGP, GOT1, IDH3G, TRAK2, PDHA1, HADH, GPT2, ACAA2, ALDH6A1, SUCLG1, FBP2, CHPT1, COX6C, NNT, AKR1B10, PGM1, ADSL, PCCB, PCCA, MDH2, MDH1, HSD17B10, ME3, NT5C1A, COX7B, ACAT1, HADHA, QRSL1, TPI1, IVD, FH, COX8B, IDH3B, DLAT, IDH3A, TST, NDUFV1, ST8SIA5, NDUFV2, ATP5D, UQCRC2, UQCRC1, CYC1, NDUFS7, MCCC2, NDUFS4, MCEE, NDUFS8, ATP5O, ATP5I, SUCLA2, NDUFS3, NDUFS2, NDUFS1, NDUFB11, ACADM, ACO2, ACADS, ALDH5A1, WBSCR17, NDUFC2, PFKM, COQ7, NDUFA10, LPIN1, NDUFA12, NDUFA11, ACADVL, COQ3, PANK1, ADK, NDUFB5, NDUFB6, NDUFB8, NFS1, ECHS1, ATP5G1, ATP5G3, ACSL1, PLCD4, ACSL6, AGL, NDUFA5, NDUFA8, NDUFA9, BCKDHB, CS, ATP5F1, SDHA, SDHB, GANC, SDHC, SDHD, GPT*	1.46E-25
Oxidative phosphorylation	*ATP5D, UQCRC2, NDUFB5, NDUFB6, UQCRC1, NDUFB8, COX7B, CYC1, ATP5G1, COX5B, ATP5G3, NDUFS7, NDUFS4, NDUFS8, ATP5O, ATP5I, NDUFS3, NDUFS2, NDUFS1, NDUFA5, NDUFB11, COX7A2, NDUFA8, COX8B, NDUFA9, NDUFC2, ATP5F1, NDUFA10, NDUFA12, NDUFA11, COX6C, SDHA, SDHB, SDHC, NDUFV1, NDUFV2, SDHD*	5.93E-23
Parkinson’s disease	*ATP5D, UQCRC2, NDUFB5, NDUFB6, UQCRC1, NDUFB8, COX7B, CYC1, PINK1, ATP5G1, COX5B, ATP5G3, NDUFS7, NDUFS4, NDUFS8, ATP5O, NDUFS3, NDUFS2, NDUFS1, NDUFA5, NDUFB11, COX7A2, NDUFA8, COX8B, NDUFA9, NDUFC2, ATP5F1, NDUFA10, NDUFA12, NDUFA11, COX6C, SDHA, SDHB, SDHC, NDUFV1, NDUFV2, SDHD*	4.58E-22
Alzheimer’s disease	*ATP5D, UQCRC2, HSD17B10, NDUFB5, NDUFB6, UQCRC1, NDUFB8, COX7B, CYC1, ATP5G1, COX5B, ATP5G3, NDUFS7, NDUFS4, NDUFS8, PPP3CB, ATP5O, NDUFS3, NDUFS2, NDUFS1, NDUFA5, NDUFB11, COX7A2, NDUFA8, COX8B, NDUFA9, NDUFC2, ATP5F1, NDUFA10, NDUFA12, NDUFA11, COX6C, SDHA, SDHB, SDHC, NDUFV1, NDUFV2, SDHD*	3.05E-20
Biosynthesis of antibiotics	*HSD17B10, ECHS1, PGAM2, OGDH, ACSS2, ACAT1, PDHB, HADHA, CMBL, PGP, ACSS1, TPI1, IDH3G, GOT1, PDHA1, SUCLA2, HADH, FH, ACAA2, ACADM, ACO2, SUCLG1, BCKDHB, CS, IDH3B, DLAT, PFKM, FBP2, IDH3A, SDHA, SDHB, SDHC, PGM1, SDHD, ADSL, PCCB, MDH2, PCCA, MDH1*	2.76E-18
Non-alcoholic fatty liver disease (NAFLD)	*UQCRC2, PPARA, NDUFB5, UQCRC1, NDUFB6, NDUFB8, COX7B, CYC1, COX5B, NDUFS7, NDUFS4, NDUFS8, PRKAA2, NDUFS3, NDUFS2, NDUFS1, NDUFA5, NDUFB11, COX7A2, NDUFA8, COX8B, NDUFA9, NDUFC2, NDUFA10, IRS1, NDUFA12, NDUFA11, COX6C, SDHA, SDHB, SDHC, NDUFV1, NDUFV2, SDHD*	8.50E-18
Huntington’s disease	*ATP5D, UQCRC2, NDUFB5, NDUFB6, UQCRC1, NDUFB8, COX7B, CYC1, ATP5G1, COX5B, ATP5G3, NDUFS7, NDUFS4, NDUFS8, ATP5O, NDUFS3, NDUFS2, NDUFS1, NDUFA5, NDUFB11, COX7A2, NDUFA8, COX8B, NDUFA9, NDUFC2, ATP5F1, NDUFA10, PPARGC1A, NDUFA12, NDUFA11, COX6C, SDHA, SDHB, SDHC, NDUFV1, NDUFV2, SDHD*	1.64E-17
Citrate cycle (TCA cycle)	*ACO2, SUCLG1, CS, IDH3B, DLAT, OGDH, IDH3A, PDHB, SDHA, SDHB, IDH3G, SDHC, SDHD, PDHA1, SUCLA2, MDH2, FH, MDH1*	3.23E-16
Valine, leucine and isoleucine degradation	*ACAA2, HSD17B10, ALDH6A1, ACADM, ACADS, BCKDHB, ECHS1, ACAT1, HADHA, HIBADH, MCCC2, IVD, OXCT1, MCEE, HADH, PCCB, PCCA*	4.13E-10

We defined three module–cluster combinations (M&Cs); the results indicated that the genes involved in modulebrown–cluster1 M&C (modulebrown&cluster1) were mainly categorized as relating to biological processes, and the top five terms were responses to stimuli including lipopolysaccharide, positive regulation of transcription from RNA polymerase II promoter, organic cyclic compound, mechanical stimulus, and cAMP. Cytokines (C–X–C motif chemokine ligand 1 (CXCL1), C–X–C motif chemokine ligand 2 (CXCL2), interleukin 6 (IL6), and interleukin 1 β (IL1B)) were produced when the tumor necrosis factor (TNF) signaling pathway was activated.

In the moduleturquoise–cluster2 M&C (moduleturquoise&cluster2), biological processes such as cell–cell adhesion, actin filament bundle assembly, receptor-mediated endocytosis, and inflammatory response were enriched, as well as genes were related to focal adhesion, endoplasmic reticulum, lysosomes etc. for cellular components and protein binding, actin filament, and cadherin binding for molecular function. A total of 13 KEGG pathways were found (*P*<0.05), of which the most important were associated with lysosomes, phagosomes, and FcγR-mediated phagocytosis. The results indicated that a number of neutrophils and macrophages clustered to remove necrotic tissue at 24–48 h post-injury, consistent with other studies [20].

The genes in the moduleblue–cluster3 M&C (moduleblue&cluster3) down-regulated at the 24–48 h stage were mainly involved in biological processes such as the tricarboxylic acid cycle, NADH metabolism, and respiratory electron transport oxidation–reduction reactions. Correspondingly, genes categorized as relating to cell components were involved in the mitochondrion, mitochondrial inner membrane, and mitochondrial respiratory chain complex I, as well as most molecular functions associated with NADH dehydrogenase (ubiquinone) activity and electron carrier activity, indicating that the oxidative phosphorylation (OXPHOS) of reduced nicotinamide adenine dinucleotide phosphate (NADPH) was greatly affected. Metabolic pathways such as carbon metabolism, OXPHOS, and fatty acid metabolism were also highly enriched.

Overall, the genes in modulebrown&cluster1 were up-regulated at 1–3 h post-injury, and they mainly responded to mechanical stimuli and triggered cellular responses to inflammation during the early (acute) phase. At the 24–48 h phase, the up-regulated genes in moduleturquoise&cluster2 related more to the inflammatory response, cell–cell adhesion, actin filament binding, inflammatory migration, and lysosomes. Phagocytosis signaling pathways (i.e., phagosome and FcγR-mediated phagocytosis pathways) important for cleaning up necrotic cellular debris were enriched, indicating that a number of inflammatory cells were attracted to the damaged area. Moreover, we found that the down-regulated genes (in moduleblue&cluster3) during the healing process at 24–48 h were largely associated with metabolic processes, especially OXPHOS of NADPH, which has been reported only rarely.

### The expression of genes regulating OXPHOS down-regulated 24–48 h after skeletal muscle contusion

**Figure 6:**
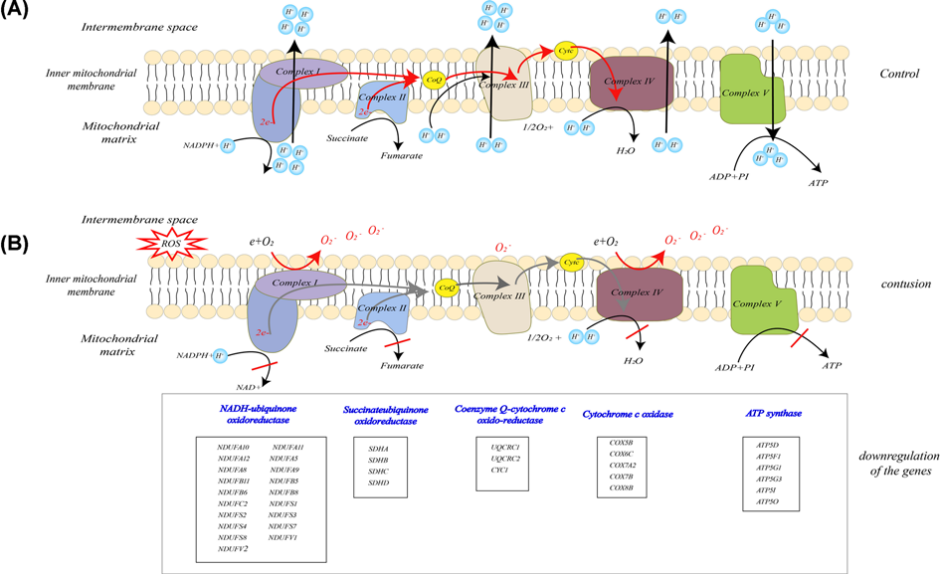
Depiction of the mitochondrial respiration chain showing the sites that generate ROS (**A**) Structural organization of the respiratory complexes in the control group. (**B**) Formation of ROS with reduced activities of complexes I–V, and a list of the down-regulated genes of each complex.

It is also notable that a number of genes down-regulated 24–48 h after the skeletal muscle was contused were related to mitochondria and enriching metabolic pathways, especially the OXPHOS pathway. After identifying the down-regulated genes associated with OXPHOS, we found that they encoded the respiratory chain of complexes I–V (Figure 6B), which act as electron carriers to generate a proton gradient on both sides of the inner mitochondrial membrane, thus driving adenosine triphosphate (ATP) synthesis (Figure 6A) [21,22]. The changes in these genes were associated with reduced activity of complexes I–V, but especially complex I, which had the largest number of down-regulated genes in the OXPHOS pathway. This might promote the generation of reactive oxygen species (ROS), given that studies have shown that inhibition of complex III (ubiquinone–cytochrome *c* reductase) increased ROS production *in vitro* [23,24], and that disruption or prevention of the interaction between complexes I and III by various means boosts the release of ROS from complex I [25,26].

During the repair process at 24–48 h, functional annotation and pathway enrichment analysis of up-regulated genes demonstrated that inflammatory cells were attracted to the damaged area to promote the healing of injured tissues, in line with other reports [13,27,28], resulting in increased levels of neutrophils and macrophages in the injured area, and increased formation of reactive species to phagocytize damaged tissues. The evidence suggests that phagocytic neutrophils generate O_2_^−^ and other oxidants using the abundant O_2_ not involved in mitochondrial respiration, through a respiratory burst catalyzed by NADPH oxidase [29,30]. Moreover, inhibition of mitochondrial complex III reduces H_2_O_2_ generation, resulting in impairment of Toll-like receptor 4-induced neutrophil activation [31]. The present study suggested that the gene expression of mitochondrial respiratory complexes down-regulated 24–48 h after trauma may be related to inflammatory cells clustering in response to increased generation of ROS, which promote phagocytosis of damaged tissues. Moreover, previous studies have shown that muscle contusion promotes mitochondrial function impairment, which is responsible for oxidative damage [32].

### PPI network analysis to identify the hub genes in each functional module and temporal cluster

**Figure 7:**
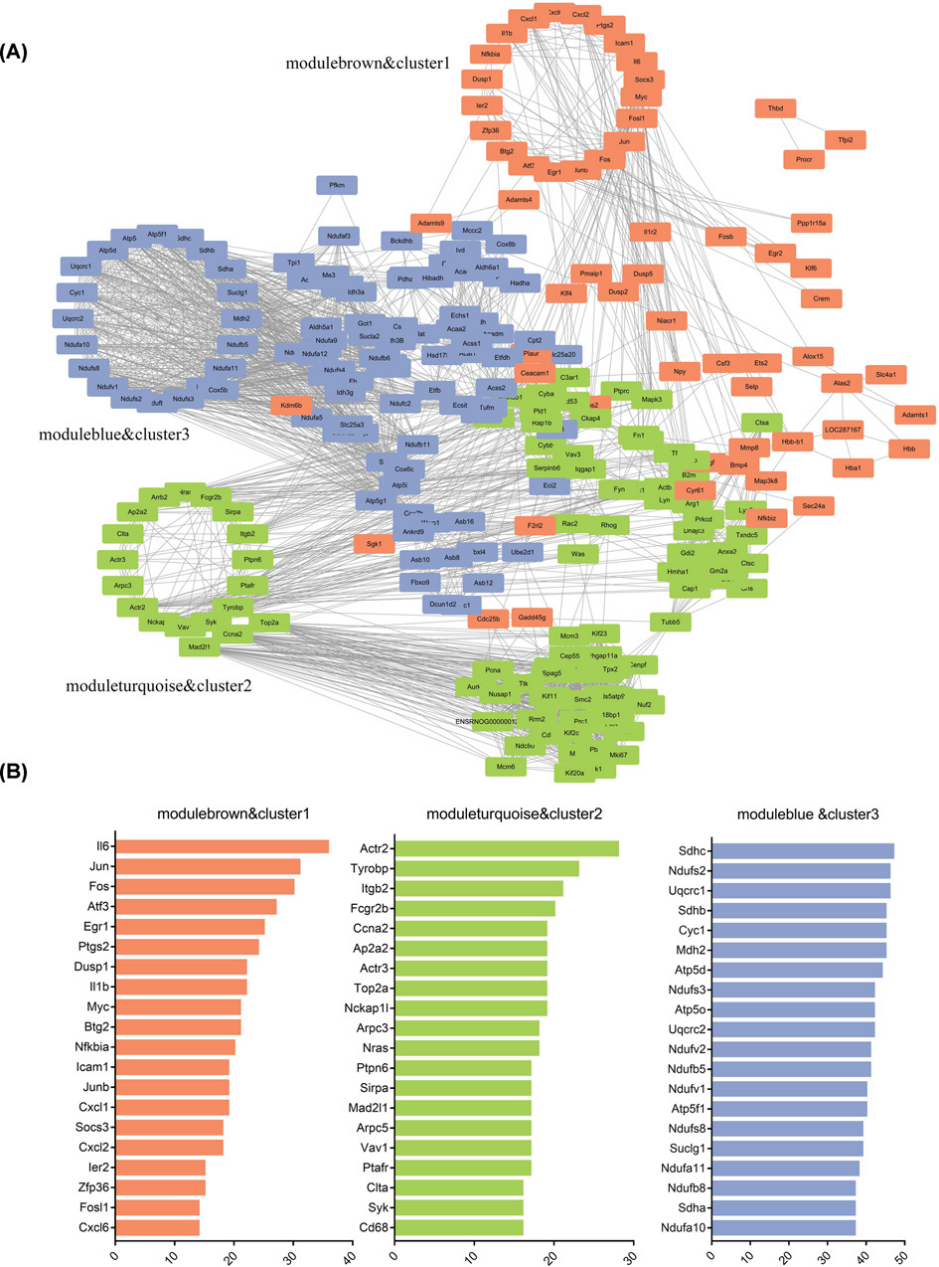
PPI network analysis of DEGs among different functional module–temporal cluster combinations (M&Cs) (**A**) Interaction network of all genes in modulebrown&cluster1 and the top 100 genes (according to degree) in the other M&Cs; the nodes for the hub genes of each M&C were arranged in a circle and the genes in each M&C had the same color. (**B**) List of hub genes for each M&C.

We constructed a PPI network to elucidate the interactions among DEGs and identify potentially crucial genes in each M&C, based on a confidence score ≥ 0.7, using the STRING database and Cytoscape software. The network was characterized by complex interplay among functional molecules. The top 20 DEGs for each M&C, in terms of the degree of connectivity, were considered as hub genes playing pivotal regulatory roles in the network. As shown in Figure 7A, the network was visualized to display the interactions among the 97 genes in modulebrown&cluster1 and the top 100 genes (according to degree of connectivity) in the other M&Cs, with the hub genes of each M&C arranged in a circle. In the network, as well as genes with high connectivity within each M&C, interactions can be seen among genes in all three M&Cs, indicating that these genes interacted during the different stages of repair after muscle injury.

The hub genes for each M&C were listed in Figure 7B; note that those in modulebrown&cluster1 were mostly involved with the TNF signaling pathway (IL6, jun proto-oncogene (Jun), and FBJ osteosarcoma oncogene (Fos)), while those in moduleturquoise&cluster2 were related to cell migration (actin-related protein 2 (Actr2), integrin subunit β 2 (Itgb2), and actin-related protein 3 (Actr3)), phagocytosis (Fc fragment of IgG receptor IIb (Fcgr2b), signal-regulatory protein α (Sirpa), and Itgb2), the inflammatory response (transmembrane immune signaling adaptor (Tyrobp), spleen-associated tyrosine kinase (Syk), and Cd68 molecule (Cd68)), and the cell cycle (DNA topoisomerase II α (Top2a), cyclin A2 (Ccna2), and mitotic arrest deficient 2 like 1 (Mad2l1)). Meanwhile, all of the hub genes for moduleblue&cluster3 encode respiratory chain complexes, confirming the relevance of the functional enrichment analysis.

## Discussion

Most of our knowledge of the muscle repair process in response to injury has been obtained through the study of muscle diseases and muscle damage associated with pathogenic processes, such as Duchenne muscular dystrophy and ischemic injury. Multiple studies have concluded that the healing process in muscle disease shares similar mechanisms to that associated with acute injury, involving damage, inflammation, regeneration, and fibrosis phases [33,34]. However, in chronic muscle diseases, in which these phases occur repeatedly and thus pathologically, there is a more complex repair response resulting in excessive accumulation of connective tissue, which can have long-term effects on muscle function [3]. Ischemic injury of skeletal muscle during surgery is a result of loss of blood flow and oxygen supply. Oxidative damage is most obvious following reperfusion, which causes an increase in ROS expression in the injured tissue [35]. Therefore, more information on the cellular and molecular events involved in mechanical muscle injury is needed, given that the transcriptional landscape of muscle contusion is not well understood. In the present study, the goal was to conduct a system-level analysis of gene expression patterns during the repair process after a muscle injury caused by mechanical stress.

A total of 3522 genes were differentially expressed during the healing process from mild and severe contusion; we further analyzed these DEGS in terms of the molecular mechanisms involved in muscle repair. Greater overlap of these DEGs (Figure 2C) between the mild and severe contusions was seen during the early stages after injury (within 48 h) compared with later stages (at 168 h), when only 29 genes were coexpressed. In the severe contusion model, at 168 h there were more up-regulated genes (552 DEGs) and a delayed induction response compared with the mild injury model, which had only 157 up-regulated DEGs. The different gene expression profiles between mild and severe models at 168 h appeared critical to the regenerative capacity, as reflected in the different muscle repair outcomes. Moreover, the results suggested common mechanisms in the transcriptomic repair of mild and severe contusions during the degeneration and early inflammatory processes, which was verified by the PCA results for the 1620 coexpressed genes.

Based on the HCL and WGCNA, 1620 genes exhibited time-dependent expression patterns, which could provide valuable information for wound age estimation. Gene expression in cluster1 was up-regulated at 1–3 h post-injury, with the genes in cluster2 also being overexpressed, while the cluster3 genes were down-regulated at 24 and 48 h post-injury. The expression patterns were confirmed by WGCNA, in which the functional modules strongly related to 1–3 and 24–48 h showed the same temporal changes (where the temporal clusters and their three corresponding functional modules had largely the same genes). These genes, the expression profiles of which not only showed strong correlations with the time of injury but were also similar between the two damage groups, are promising markers for wound aging.

In cases of violent injury, the severity of muscle bruising varies among individuals, and among sites of damage within the same individual, which presents a challenge for forensic pathologists in determining the time of injury. Identifying similarities in the cellular response and molecular composition of the microenvironment during muscle repair following damage varying in severity is of fundamental importance for accurate determination of wound age in forensic practice. Some studies performed microarray analysis to identify time-dependent gene expression patterns [36], while others characterized the time course of the expression of several genes by quantitative polymerase chain reaction to estimate wound age [15,37]. However, whether gene expression patterns are the same regardless of injury severity remains unclear. In the present study, we discovered that functional modules and temporal clusters had similar expression profiles following mild and severe muscle injury, which allowed us to identify markers of wound age.

Through gene set enrichment and PPI network analyses, the biological functions, key pathways, and hub genes involved in each M&C were characterized (see Figures 5 and 7). We found that the genes up-regulated at 1–3 h post-injury were mainly GO biological processes related to mechanical stress and cellular responses to inflammation. Also, the hub genes identified in the PPI network mostly related to the TNF signaling pathway, such as IL6, Cxcl1, and Cxcl2, which produce cytokines and chemokines that promote leukocyte recruitment and migration [38,39]. In moduleturquoise&cluster2, the genes were more associated with the inflammatory response, cell–cell adhesion, and actin filament binding, which promote inflammatory cell migration and lysosomes. Phagocytosis signaling pathways (i.e., phagosome and FcγR-mediated phagocytosis pathways) were enriched. There is strong evidence that muscle injury typically initiates rapid invasion of the damaged site by neutrophils and macrophages [7,27]. Morphological observations indicated that the number of macrophages reach a peak approximately 2–3 days after damage [20], while neutrophils accumulate at the site of injury within hours and predominate at 24 h [13]. Although some studies have showed that inflammation severity varies depending on the extent and type of damage [40]; based on our data, it seems that the damage-associated molecules released by disrupted myofibers, and the molecular mechanisms underlying inflammatory cell migration to the injury site and phagocytosis of necrotic debris, are relatively similar between mild and severe forms of muscle contusion.

Interestingly, we found that genes involved in metabolic processes, especially OXPHOS, were significantly down-regulated during the repair process at 24-48 h. The genes relating to cell components that showed changes in expression were mostly associated with mitochondria, which as the central organelle plays an indispensable role in energy metabolism, using chemical energy to produce ATP [31,41]. As known, skeletal muscle fibers contain large numbers of mitochondria for ATP synthesis through OXPHOS [42]. It has been reported that genes involved in mitochondrial OXPHOS and related functions were down-regulated after burn injury in skeletal muscle. Also, Jin et al. [43] showed that DEGs down-regulated at 2 weeks after spinal cord injury were mainly involved in OXPHOS, while Baek et al. [44] found that mitochondrial functioning was impaired in the acute phase (3 h) after spinal cord injury. These studies suggest that injury could induce changes in metabolic and mitochondrial pathways, but there are few data regarding these changes in skeletal muscle after contusion.

OXPHOS, the most obviously down-regulated pathway, is required for ATP synthesis, which involves generating a proton gradient on both sides of the inner mitochondrial membrane, and is also the main source of ROS [45]. The reduced expression of oxidation- and respiratory complex-related genes probably resulted in impaired mitochondrial respiration and the production of a huge quantity of ROS at 24–48 h after muscle injury (Figure 6). Several lines of evidence suggest that the mitochondrial OXPHOS system is the primary site of action for acute inflammation, in association with enhanced ROS generation from local cell populations, as well as neutrophil and macrophage accumulation at the damaged site [46]. The removal of tissue debris by phagocytes is a common cellular mechanism that occurs in the early stage following muscle injury; our findings support this [3]. Excessive production of ROS during phagocytic and inflammatory processes should exacerbate muscle damage, which in turn exacerbates mitochondrial function impairment and leads to the generation of more ROS. Thus, it is necessary to control the intensity of the inflammatory response to reduce oxidative damage, which also results from ROS overproduction. It seems that the 24–48 h post-injury period, when inflammatory cells clearly responded, may be the best time for treatment of oxidative damage. While the origin of oxidative damage and metabolic changes following muscle contusion are not fully understood, our findings may serve as reference data aiding the identification of new targets for the treatment of muscle injury.

In the present study, we used microarray technology to provide a comprehensive summary of the genes involved in skeletal muscle repair in rats. Our results suggest that the molecular mechanisms underlying cellular functions were similar within 48 h post-contusion between the mild and severe contusion groups. Moreover, interestingly, we found that the genes associated with metabolic processes, especially OXPHOS, were significantly down-regulated at 24–48 h after injury. Understanding this phenomenon, which has been reported only rarely, may yield new information pertinent to muscle repair and warrants further investigation. Furthermore, the genes with time-dependent expression pattern in M&C showed similar expression profiles following mild and severe muscle contusion, and should aid in the estimation of wound age in forensic practice.

## Conclusions

Current understanding of healing cascades following muscle contusion, and how they potentially determine outcomes, is incomplete. Our comprehensive analysis of transcriptional data for two muscle damage groups differing in severity provides new insight into the molecular mechanisms underlying muscle repair. However, there remains translational challenges. Further studies are required according to the limitations of animal experiments which it always raises the question whether it can be transferred to human conditions. Due to the sample size for microarray analysis was small, the obtained genes in the present study need to be verified using other methods of future research.

## Data Availability

The microarray data in the present study have been upload in GEO (accession number: GSE162565). Other data generated or analyzed during the present study are included within the supplementary information files.

## Abbreviations

ATP: adenosine triphosphate
CXCL1: C–X–C motif chemokine ligand 1
CXCL2: C–X–C motif chemokine ligand 2
DEG: differentially expressed gene
FDR: false discovery rate
GO: Gene Ontology
GS: gene significance
HCL: hierarchical clustering
IL6: interleukin 6
Itgb2: integrin subunit β 2
KEGG: Kyoto Encyclopedia of Genes and Genomes
M&C: module–cluster combination
ME: module eigengene
MM: module membership
NADPH: nicotinamide adenine dinucleotide phosphate
OXPHOS: oxidative phosphorylation
PCA: principal component analysis
PPI: protein–protein interaction
ROS: reactive oxygen species
sscDNA: sense-strand cDNA
Syk: spleen-associated tyrosine kinase
TNF: tumor necrosis factor
WGCNA: weighted gene co-expression network analysis
